# A Biostimulant Obtained from the Seaweed *Ascophyllum nodosum* Protects *Arabidopsis thaliana* from Severe Oxidative Stress

**DOI:** 10.3390/ijms21020474

**Published:** 2020-01-11

**Authors:** Mohammad Amin Omidbakhshfard, Neerakkal Sujeeth, Saurabh Gupta, Nooshin Omranian, Kieran J. Guinan, Yariv Brotman, Zoran Nikoloski, Alisdair R. Fernie, Bernd Mueller-Roeber, Tsanko S. Gechev

**Affiliations:** 1 Max Planck Institute of Molecular Plant Physiology, Am Mühlenberg 1, 14476 Potsdam-Golm, Germany; omidbakhshfard@mpimp-golm.mpg.de (M.A.O.); gupta@uni-potsdam.de (S.G.); omranian@mpimp-golm.mpg.de (N.O.); brotman@mpimp-golm.mpg.de (Y.B.); fernie@mpimp-golm.mpg.de (A.R.F.); mueller@mpimp-golm.mpg.de (B.M.-R.); 2 BioAtlantis Ltd., Clash Industrial Estate, Tralee, V92 RWV5 Co. Kerry, Ireland; research@BioAtlantis.com; 3 Molecular Biology, Institute of Biochemistry and Biology, University of Potsdam, Karl Liebknecht Str. 24-25, 14476 Potsdam-Golm, Germany; 4 Bioinformatics, Institute of Biochemistry and Biology, University of Potsdam, Karl Liebknecht Str. 24-25, 14476 Potsdam-Golm, Germany; nikoloski@uni-potsdam.de; 5 Department of Molecular Stress Physiology, Center of Plant Systems Biology and Biotechnology, 139 Ruski blvd., 4000 Plovdiv, Bulgaria; tsangech@uni-plovdiv.bg; 6 Department of Plant Physiology and Molecular Biology, University of Plovdiv, 24 Tsar Assen Str., 4000 Plovdiv, Bulgaria

**Keywords:** *Ascophyllum nodosum*, *Arabidopsis thaliana*, biostimulant, paraquat, priming, oxidative stress tolerance, reactive oxygen species

## Abstract

Abiotic stresses cause oxidative damage in plants. Here, we demonstrate that foliar application of an extract from the seaweed *Ascophyllum nodosum*, SuperFifty (SF), largely prevents paraquat (PQ)-induced oxidative stress in *Arabidopsis thaliana*. While PQ-stressed plants develop necrotic lesions, plants pre-treated with SF (i.e., primed plants) were unaffected by PQ. Transcriptome analysis revealed induction of reactive oxygen species (ROS) marker genes, genes involved in ROS-induced programmed cell death, and autophagy-related genes after PQ treatment. These changes did not occur in PQ-stressed plants primed with SF. In contrast, upregulation of several carbohydrate metabolism genes, growth, and hormone signaling as well as antioxidant-related genes were specific to SF-primed plants. Metabolomic analyses revealed accumulation of the stress-protective metabolite maltose and the tricarboxylic acid cycle intermediates fumarate and malate in SF-primed plants. Lipidome analysis indicated that those lipids associated with oxidative stress-induced cell death and chloroplast degradation, such as triacylglycerols (TAGs), declined upon SF priming. Our study demonstrated that SF confers tolerance to PQ-induced oxidative stress in *A. thaliana*, an effect achieved by modulating a range of processes at the transcriptomic, metabolic, and lipid levels.

## 1. Introduction

Oxidative stress in plants occurs as a consequence of many abiotic stresses including extreme temperatures, drought, salinity, osmotic stress, and pollutants (e.g., heavy metals or herbicides) [1,2,3,4,5]. Abiotic stress-induced oxidative stress, manifested as an increased production of reactive oxygen species (ROS), has detrimental effects on plant growth, inhibits photosynthesis, leads to cell damage, and, in the most severe cases, results in the activation of programmed cell death [6]. The herbicide paraquat (PQ) causes oxidative stress by accepting electrons from the reduced end of photosystem I and transferring them to molecular oxygen, thereby generating superoxide radicals, which in turn are converted to hydrogen peroxide by superoxide dismutases (SODs) [7,8]. Paraquat is widely used to study the effects of oxidative stress to isolate mutants and investigate the functions of genes involved in ROS detoxification pathways [7,9,10,11,12,13]. In addition to SODs and other antioxidant enzymes that are part of the elaborate antioxidant defense network, plants employ various molecular strategies to counteract oxidative stress and minimize its detrimental effect, including the accumulation of osmoprotectants and compatible solutes, stress protective proteins such as late embryogenesis abundant (LEA) proteins/dehydrins, heat shock proteins (HSPs), and an activation of cellular repair mechanisms [8,14,15].

Molecular priming is a phenomenon by which biologically active molecules activate plant defense mechanisms and secure tolerance against a subsequent stress. For example, application of hydrogen peroxide (H_2_O_2_) at low doses can lead to tolerance against oxidative stress induced during chilling, high light, and toxicity due to the presence of heavy metals and pathogens in many plant species [5,16,17,18,19,20]. Molecular priming is similar to the process of acclimation, where application of a mild stress, for example, at a low temperature can protect plants against subsequent, more severe stress events (e.g., chilling/freezing) [3,4]. Both molecular priming and acclimation alter transcriptomes and metabolomes, resulting in molecular re-adjustments that are necessary and sufficient to provide stress protection [21]. Although not deeply studied yet, a re-adjustment of the lipidome is an important part of the metabolome that captures the entirety of lipids in a biological system [22,23].

“Biostimulants” or “plant strengtheners” are considered metabolic enhancers other than fertilizers and are being used worldwide to enhance crop resistance to various stresses while also enhancing plant growth and performance [24,25,26,27]. A wide range of biostimulants are commercially produced using brown seaweed species such as *Ascophyllum nodosum* as a raw material [28]. However, based on the extraction methodology used and the quantity and availability of bioactives present in the extracts, they can exert different effects with respect to plant growth and stress mitigation [28,29]. SuperFifty^®^ (SF) is a commercially available biostimulant which contains highly concentrated *A. nodosum* extract (500 g/L) produced in an extraction process at high temperature and pressure [29]. Experiments using SF have shown its effectiveness in reducing abiotic stress and increasing plant growth [29]. However, the molecular mode of action that leads to improved stress tolerance is currently unknown. One possibility is that different bioactive compounds present in *A. nodosum* extracts work synergistically to modulate innate pathways for biosynthesis of endogenous phytohormones. This may influence developmental processes, resulting in increased photosynthetic efficiency and delayed senescence in treated crops [30,31,32]. *Ascophyllum* extracts contain a range of organic compounds such as laminarin, fucoidan, polyphenols, mannitol, and alginates [33]. These diverse molecules including uncommon or unique polysaccharides could potentially prime and elevate stress tolerance in plants and improve plant growth. *Ascophyllum* extracts can reduce drought stress in tomato [34] and alleviate drought and salinity stress in *Arabidopsis* [31,35]. An in-depth understanding of the molecular mechanisms through which these extracts induce stress tolerance is required for accurate applications in agriculture. 

We hypothesized that SF increases abiotic stress tolerance in plants by inducing pathways associated with the reduction of oxidative stress. To test this hypothesis, the efficacy of SF was examined in a PQ-induced oxidative stress model in *Arabidopsis thaliana*, involving an in-depth “OMICs” characterization of the responses of treated plants at transcriptome, metabolome, and lipidome levels. We showed that SF markedly increased tolerance to oxidative stress in *Arabidopsis*. This was accompanied by transcriptome reprogramming and metabolome (including lipidome) readjustments, resulting in the upregulation of defense genes, repression of cell death-associated genes, and increased levels of stress-protective metabolites.

## 2. Results and Discussion

### 2.1. SF Protects Arabidopsis from Oxidative Stress

**Figure 1:**
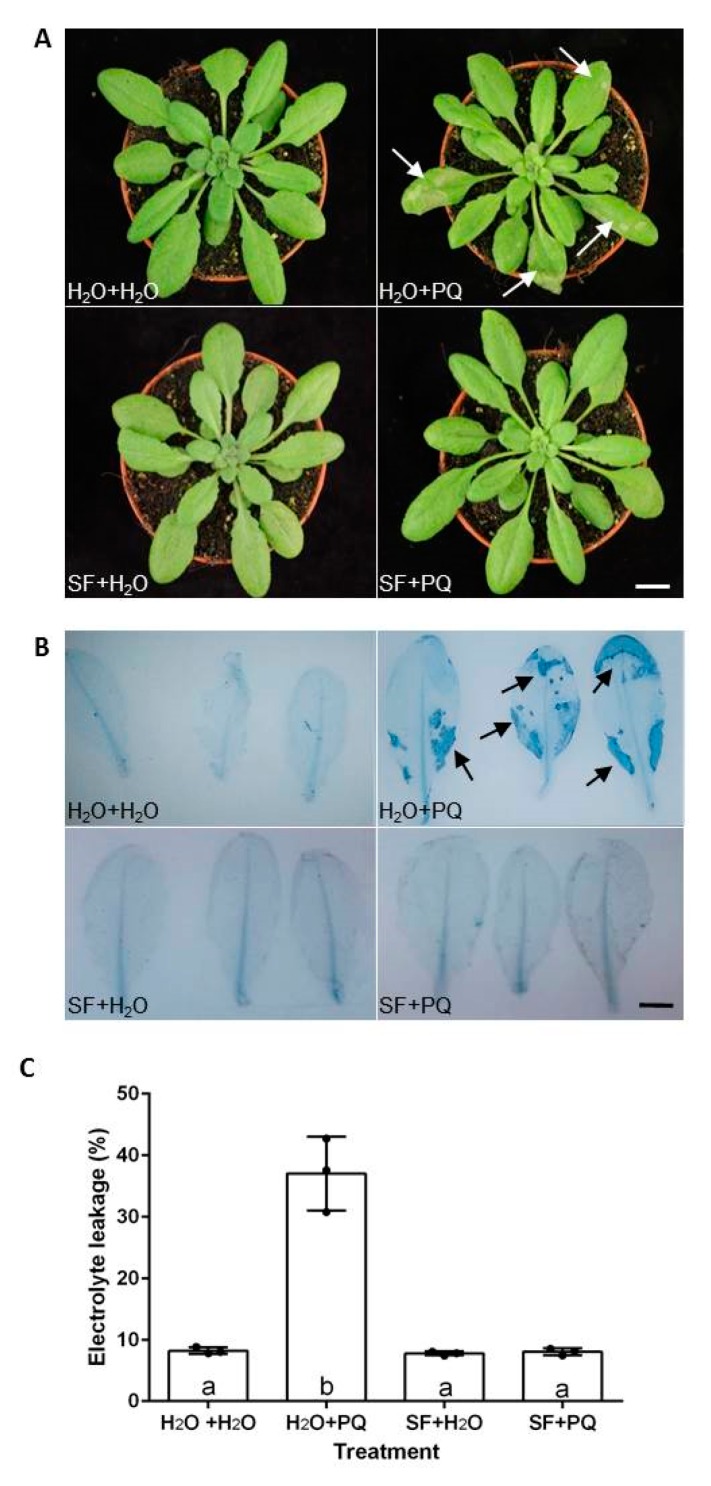
*Arabidopsis* tolerates oxidative stress upon SuperFifty (SF) priming. (**A**) Rosette phenotype of four-week-old plants pretreated and treated, respectively, with H_2_O and H_2_O (unprimed and unstressed), H_2_O and PQ (paraquat; oxidatively stressed), SF and H_2_O (SF-primed, but unstressed), and SF and PQ (SF-primed and oxidatively stressed); for details, see Materials and Methods. (**B**) Trypan blue stained leaves of the plants from panel (**A**). Blue-stained regions (arrows) of leaves in the top-right panel show dead cells after PQ (15 µM) treatment; such cell death areas are not visible in leaves primed with SF and then treated with PQ (bottom right panel). Leaves are representative examples from 30 plants in each condition. Scale bar is 10 mm and 5 mm in panels (**A**) and (**B**), respectively. (**C**) Cell death quantified by measuring electrolyte leakage from leaves. Conductivity was determined 48 h after incubation of two leaves from individual rosettes in 25 mL deionized water for 12 h. Mean values are averages of three independent experiments with each point on the graph representing individual experimental replicates. In each experiment, ten rosettes were analyzed per treatment. Mean values were compared between groups by applying one-way ANOVA followed by Tukey’s multiple comparisons test. An absence of letter sharing among the treatment groups denotes a statistically significant difference among those groups (*p* < 0.0001). Error bars denote standard deviation (SD). Full data are given in Supplementary Materials Data S1.

Oxidative stress was induced by spraying twenty-three-day-old *A. thaliana* Col-0 plants with 15 µM PQ (Figure 1). Only PQ-treated plants developed large visible necrotic lesions within one day after treatment (Figure 1A). The presence of cell death was demonstrated by trypan blue staining (Figure 1B). To investigate the protective role of SF, *Arabidopsis* plants were pre-treated or primed with 0.1% SF six times on two consecutive days prior to the day of the PQ treatment. The SF-primed plants subjected to the subsequent PQ treatment did not develop any necrotic lesion on the leaves (Figure 1A), and trypan blue staining did not detect dead cells (Figure 1B), demonstrating that SF fully protects the plants from PQ-induced oxidative cell death. Cell death was further quantified by monitoring the electric conductivity of solutions in which rosette leaves from treatment groups and untreated controls were immersed (Figure 1C; Supplementary Materials Data S1). Severe ion leakage was observed in PQ-treated plants, likely due to the ROS-induced oxidative damage [36]. However, when SF-primed plants were challenged with PQ, no increase in ion leakage was observed. Our results demonstrate that SF priming improved cell membrane integrity, reducing cell death induced by PQ.

### 2.2. RNA-Seq Identifies Genes and Pathways Associated with SF-Induced Oxidative Stress Protection

By comparing gene expression in the different samples, we identified a total of 2192 significantly differentially expressed genes (DEGs) (Supplementary Materials Data S2; see Materials and Methods for details). To identify changes at the gene expression level, differential expression analysis was performed for a number of pairwise combinations (Table S1). In general, when PQ was applied alone, significant changes were observed in the expression of genes compared to the untreated control (H_2_O + H_2_O); the vast majority of the genes affected by PQ treatment (>1400) were upregulated (>1200). Pre-treatment with SF (“priming”) prior to the PQ application resulted in a substantially lower number of DEGs (317) compared to untreated controls. Importantly, SF-primed PQ-stressed plants exhibited only a very small number of significant changes compared to plants treated with SF alone (six genes). Application of SF alone led to significant changes of only 163 genes compared to untreated controls. Our results suggest that SF primes plants in a manner which prevents PQ from inducing large-scale molecular or phenotypic changes (see Figure 1 for comparison). While SF induces only limited molecular or phenotypic changes when applied to unstressed plants, the biological effects of SF are striking when examined in the context of applying an oxidative stress-inducing agent such as PQ.

#### 2.2.1. Cluster Analysis of DEGs

The DEGs from all pairwise comparisons were combined and clustered into 20 clusters using k-means clustering (Figure S1). These 20 coordinated expression patterns revealed gene modulation and key transition states due to the treatments. Clusters 4, 7, 19, and 20 consisted of genes downregulated due to the SF priming in PQ-stress and non-stress conditions. Cluster 6 consisted of genes upregulated upon SF priming in PQ-stress and non-stress conditions. The remaining clusters exhibited upregulated genes (clusters 2, 3, 5, 8, 9, 10, 13, 16, 17, and 18) or downregulated genes (clusters 1, 11, and 15) due to the PQ induced oxidative stress. Interestingly, genes belonging to clusters that were up- or downregulated by PQ were not affected when plants were primed with SF prior to PQ application. Thus, treatment with SF prevented alteration of these genes that would otherwise occur due to the PQ application (Figure S1). Expression levels and log_2_ fold changes among treatments for the DEGs in each cluster are given in Supplementary Materials Data S2.

#### 2.2.2. Gene Set Enrichment Analysis of DEGs 

To gain insights into the molecular mechanisms involved in SF-mediated stress tolerance, we performed a Gene Ontology (GO) enrichment analysis of the DEGs (Supplementary Materials Data S3). In non-stress conditions, 128 genes were significantly downregulated upon SF priming versus the untreated control with the following GO terms being enriched: “trehalose metabolism in response to stress” (GO:0070413), “regulation of proteolysis” (GO:0030162), “response to chitin” (GO:0010200), “response to oxidative stress” (GO:0006979). Thirty-five DEGs were significantly upregulated in SF-primed versus unprimed control plants (Table S2), including several ERF/AP2 transcription factors such as *ERF15*, a positive regulator of ABA responses [37], *ERF34*, a transcriptional regulator of primary cell wall biosynthesis [38], *BBX30*, a B-box-type zinc finger protein involved in developmental regulation in long day-grown plants [39], and *TINY2*, a potential regulator of genes in the response to environmental stresses [40]. Also, *CIPK20*, which encodes a CBL-interacting protein kinase, was specifically upregulated in SF-primed plants. CIPK20, also known as PKS18, is involved in abscisic acid (ABA) signaling; its inhibition by RNA interference leads to ABA insensitivity [41]. Other genes specifically upregulated upon SF priming are: *WAG1* encoding a serine/threonine-protein kinase involved in auxin signaling and the regulation of differential growth, tissue patterning, and organogenesis [42], and *SPX1*, a gene encoding a protein involved in responses to environmental cues and internal regulation of nutrition homeostasis [43]. A gene with unknown function (*AT2G19650*) coding for a putative cysteine/histidine-rich C1 domain family protein and several other unknown proteins encoded by genes *AT3G44450*, *AT5G66740*, *AT2G15020*, *AT2G44940*, *AT5G11590*, *AT3G45210*, *AT1G04570*, *AT5G61570*, and *AT3G04140* were also specifically upregulated upon SF priming compared to PQ-treated plants and untreated controls (Supplementary Materials Data S2).

**Figure 2:**
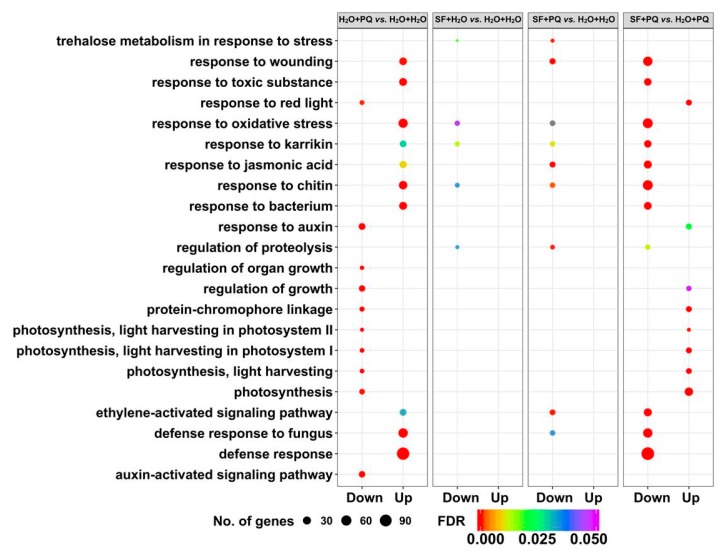
Comparison of the top five enriched GO categories from all pairwise combinations. The top five (most significant) enriched biological process GO terms from all pairwise combinations are illustrated as a bubble plot. The size and color of the circle denotes the number of differentially expressed genes and FDR (false discovery rate) adjusted *p*-value, respectively. Unprimed and unstressed (H_2_O + H_2_O), PQ-stressed (H_2_O + PQ), SF-primed but unstressed (SF + H_2_O), and SF-primed and stressed (SF + PQ).

The top five enriched GO terms from each pairwise comparison, based on FDR (false discovery rate) values, were then selected and compared between the pairwise combinations. This analysis demonstrates that enrichment and transition of key biological processes and functions occurred during stress and upon SF priming (Figure 2). The abiotic stress-related GO terms “response to oxidative stress”, “response to wounding”, and “response to toxic substance” were upregulated after PQ stress. In stark contrast, SF priming resulted in the downregulation of these stress-associated GO terms when subsequently exposed to PQ. Priming with SF specifically upregulated GO terms related to photosynthesis, hormones, and plant growth such as “response to red light” (GO:0010114), “protein–chromophore linkage” (GO:0018298), “photosynthesis” (GO:0015979), “light harvesting in photosystem I” (GO:0009768), “light harvesting in photosystem II” (GO:0009769), “response to auxin” (GO:0009733), and “regulation of growth” (GO:0040008) when exposed to PQ stress. It is also interesting that biotic stress and defense-related responses such as “response to chitin” (GO:0010200), “response to bacterium” (GO:0009617), “defense response to fungus” (GO:0050832), and “ethylene-activated signaling pathway’” (GO:0009873) terms were downregulated after SF priming followed by subsequent exposure to PQ. This supports our previous findings that a significant degree of specificity exists for extracts of *A. nodosum* in terms of stress reduction with efficacy clearly related to temperatures involved in the extraction process, i.e., high-temperature derived extract (SF) is effective in enhancing growth in abiotic stress condition compared to other lower-temperature derived extracts [29].

**Figure 3:**
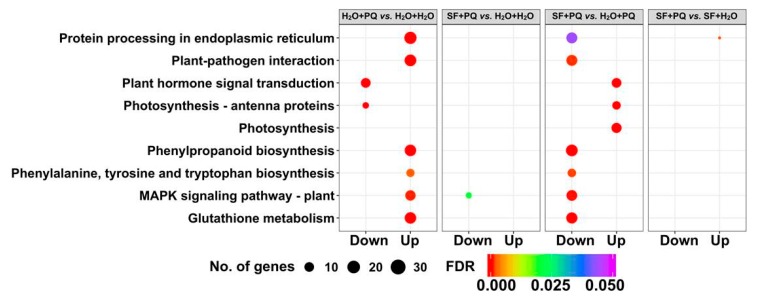
Comparison of the top five enriched KEGG (Kyoto Encyclopedia of Genes and Genomes) pathways. The top five (i.e., most significant, enriched) KEGG pathways from all pairwise combinations are illustrated as a bubble plot. The size and color of the circle denotes the number of differentially expressed genes and FDR adjusted *p*-value, respectively. Unprimed and unstressed (H_2_O + H_2_O), PQ-stressed (H_2_O + PQ), SF-primed but unstressed (SF + H_2_O), and SF-primed and stressed (SF + PQ).

To obtain a complete view of significantly altered pathways at the transcriptional level during PQ treatment and upon SF priming, we performed a KEGG (Kyoto Encyclopedia of Genes and Genomes) pathway analysis [44] for DEGs from different pairwise comparisons (Figure 3). The following pathways were significantly upregulated in PQ-induced stress conditions: “protein processing in endoplasmic reticulum” (ko04141), “plant-pathogen interaction” (ko04626), “phenylpropanoid biosynthesis” (ko00940), “phenylalanine, tyrosine, and tryptophan biosynthesis” (ko00400), “MAPK signaling pathway–plant” (ko04016), and “glutathione metabolism” (ko00480). Notably, these pathways were significantly downregulated in SF-primed PQ-stress condition. On the other hand, SF priming followed by PQ treatment specifically upregulated pathways for “plant hormone signal transduction” (ko04075) and “photosynthesis” (ko00195).

**Figure 4:**
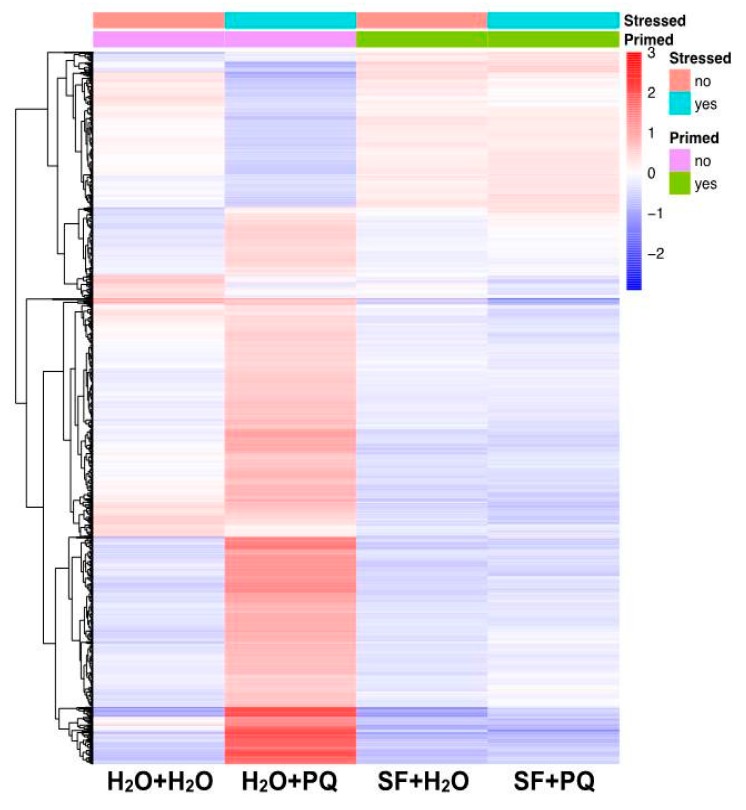
Transcriptional regulation upon PQ stress in SF-primed and unprimed *Arabidopsis* plants. Heatmap of all differentially expressed genes. Color scale represents the mean centered log_2_ normalized TMM values (Trimmed Mean of M values) averaged across three biological replicates. Full data are given in Supplementary Materials Data S1 and S2.

Overall, the transcriptome analysis in this study demonstrated that PQ-induced oxidative stress causes extensive transcriptional reprogramming as evidenced by the upregulation of many stress marker genes, downregulation of many photosynthesis- and growth-related genes, and induction of genes involved in cell death. Upregulation of stress genes and downregulation of photosynthesis and growth genes are hallmarks of severe stress and cell death [45,46]. Such an extensive level of transcriptional reprogramming was absent in the SF-primed plants exposed to PQ, with no induction of stress genes and no downregulation of photosynthesis and growth genes. The overall pattern of differential gene expression between untreated, PQ stressed, SF priming, and SF priming PQ-stress treatments are represented by a heatmap (Figure 4). The heatmap demonstrates that at the transcriptome level, plants subjected to PQ stress after pre-treatment with SF resemble SF-primed and unstressed plants.

#### 2.2.3. Annotation of Genes Induced by SF for Oxidative Stress Protection

Based on the enrichment analysis, DEGs were selected from the various treatments (Table 1) for an in-depth literature review of the known functions of these genes. This investigation revealed genes that could be directly or indirectly involved in SF priming-induced oxidative stress tolerance. It also identified potential adaptions possibly underlying the stress tolerance phenotype.

##### ROS-Responsive Genes, Programmed Cell Death, and Senescence 

In a previous study, we identified five genes that are hallmarks for oxidative stress [47]; each of them was highly induced by PQ treatment (Table 1). Besides the ROS inducible/marker genes [47], Table 1 contains the genes most regulated by PQ or/and SF that represent pathways and processes such as ROS dependent PCD and senescence, ROS detoxification, photosynthesis, carbohydrate metabolism and cellulose synthesis, growth and hormone signaling, autophagy, lipid metabolism, transcription factors, and stress-related genes. In addition, 140 out of 177 genes, known to be ROS responsive [48], were induced by PQ treatment (Supplementary Materials Data S4). Our results therefore indicate that PQ treatment caused prominent oxidative stress similar to previous reports [47,49,50]. In contrast, none of these genes were upregulated in SF-primed PQ-treated plants (Supplementary Materials Data S4).

Reactive oxygen species can induce programmed cell death (PCD) and metacaspases play a key role in several types of ROS-induced PCD [51]. Two metacaspase-encoding genes, *AtMC2* and *AtMC8*, were highly induced by PQ in the unprimed plants but not in the SF-primed plants. Moreover, *PROMOTION OF CELL SURVIVAL 1* (*PCS1*), encoding an aspartic protease that regulates cell death and promotes cell survival, was induced in both SF-primed and primed/stressed plants (Table 1). *PCS1* plays an important role in reproductive gametogenesis and its loss-of-function mutation causes excessive cell death of embryonic tissues [52]. The above results demonstrate that SF prevents the activation of the ROS-triggered PCD pathway. In addition, we observed an upregulation of *SENESCENCE-SUPPRESSED PROTEIN PHOSPHATASE* (*SSPP*) and a downregulation of the transcription factor *RD26* as well as of *SENESCENCE-ASSOCIATED GENE 13* (*SAG13*) in SF-primed and primed/stressed plants (Table 1). SSPP functions in sustaining leaf longevity and its overexpression significantly delays leaf senescence in *Arabidopsis* [53]. The transcription factor RD26 (ANAC072) is a positive regulator of stress- and darkness-induced senescence [54,55]. As we recently demonstrated, an enhanced expression of *RD26* triggers the accumulation of free amino acids (in particular Gln and Asn), enhances the accumulation of tricarboxylic acid (TCA) cycle intermediates and represses the accumulation of γ-aminobutyric acid (GABA; [54]). *SAG13* is induced by stresses such as darkness, drought, wounding, and pathogen challenge in leaves [56] and is involved in the breakdown of cellular components during stress-induced senescence. Expression of *SAG13* is directly controlled by RD26 [54]. The induced expression of both, *RD26* and *SAG13* by PQ and repression of these genes by SF priming, therefore, is likely to contribute to the absence of cell death in SF-treated plants (Table 1).

##### Detoxification of PQ-induced ROS

**Figure 5:**
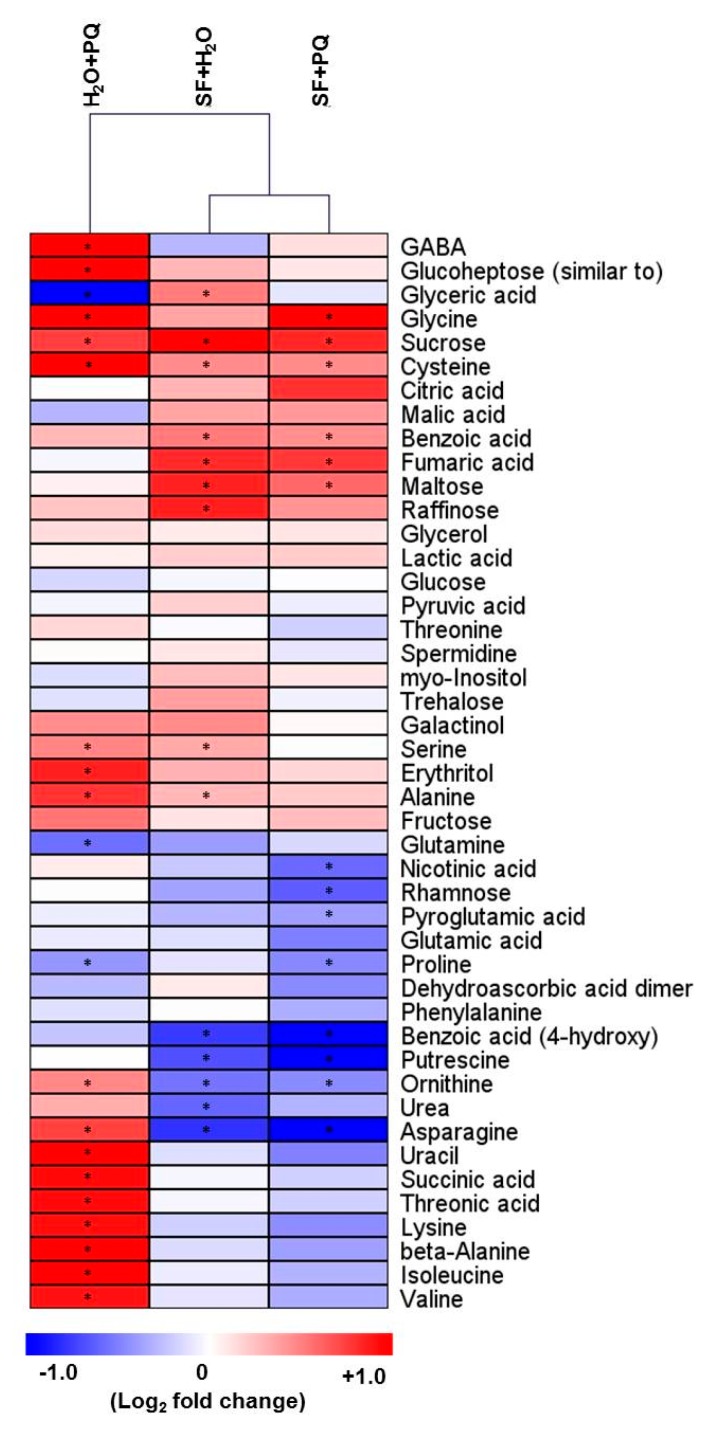
Primary metabolite changes induced by oxidative stress. Changes in primary metabolite abundances of *Arabidopsis* leaves upon foliar spray with PQ and/or SF measured by GC-MS. Blue and red depict a decrease and increase, respectively (log_2_ fold change), compared to the control (unprimed and unstressed, i.e., H_2_O + H_2_O). The data are averages of seven biological replicates. Samples: PQ-stressed (H_2_O+PQ), SF-primed, but unstressed (SF + H_2_O), and SF-primed and stressed (SF + PQ). Statistically significant differences to control are highlighted by asterisks (Student’s *t*-test, * *p*-value < 0.05). Full data are given in Supplementary Materials Data S5.

PQ produces superoxide radicals, followed by a cascade of reactions producing hydrogen peroxide and hydroxyl radicals [57,58]. Accumulation of these toxic free radicals will result in a decline of photosynthesis, an increase of lipid peroxidation and the destruction of cell membranes, leading to chlorosis and necrosis of plant tissues [58,59]. With respect to enzymatic antioxidants, SF-primed plants had higher levels of *Fe SUPEROXIDE DISMUTASE 1* (*FSD1*) and *ASCORBATE PEROXIDASE 1* and *4* (*APX1* and *APX4*) compared to plants stressed with only PQ (Table 1). Increased expression of these genes can assist the detoxification of superoxides and peroxides [60,61,62]. In addition, higher levels of *AtOXR*, which encodes transmembrane OXR protein, was observed in SF-primed plants (Table 1). Overexpression of *AtOXR* in *Arabidopsis* results in ascorbate accumulation, concomitant with an increased tolerance to oxidative stress [63]. Notably, expression of 1-*myo*-inositol-1-phosphate synthase encoding *MIPS1* was elevated in SF-primed plants. MIPS catalyzes the rate-limiting step in the synthesis of *myo*-inositol which is critical for maintaining cellular levels of ascorbic acid [64]. In addition to transcriptome profiling, we also analyzed the primary metabolic profiles using GC-MS (see below). We found an increased level of threonate in PQ-stressed plants, an effect that was entirely mitigated when plants were primed with SF prior to the PQ treatment (Figure 5). Threonate typically accumulates due to the degradation of ascorbate during stress, which appears to be suppressed by SF. In accordance with the strong priming effect of SF, the gene coding for L-ascorbate oxidase (AO) that converts ascorbate to dehydroascorbate (DHA) had higher expression in SF-primed plants (Table 1). DHA is transported from the apoplast to the cytosol in exchange with ascorbate (reduced form), to ensure a constant apoplast redox flux [65,66,67,68]. The antioxidant ascorbate is one of the key components for reducing ROS. It also acts as a redox buffer and regulates the response to the extracellular environment in the apoplast [64]. The above observations suggest that priming by SF treatment induces a ROS detoxification pathway that involves the antioxidant ascorbic acid.

A gene coding for an iron–sulfur domain containing protein, *NEET*, was also upregulated in SF-primed plants (Table 1). This gene has a role in regulating redox reactions and the control of apoptosis. In *Arabidopsis*, T-DNA knockdown and RNA interference *NEET* lines accumulated higher levels of ROS than wild-type control plants [69]. NEET is involved in iron (Fe) homeostasis, supporting a proper ROS balance and photosynthesis in cells [70,71]. Notably, several cytochrome P450 encoding genes (*CYP76C1*, *CYP71B35,* and *CYP706A*) were also more strongly expressed in SF-primed plants (Table 1). Cytochrome P450 enzymes have previously been shown to be involved in herbicide metabolism and detoxification [72,73].

##### Photosynthetic Genes

Most of the photosynthesis-related genes were significantly downregulated by PQ in unprimed plants. In fact, this is the largest functional group of genes that was repressed by PQ. The group included genes encoding subunits of photosystem II, genes involved in chlorophyll biosynthesis, such as protochlorophyllide oxidoreductase, genes from the Calvin–Benson cycle such as RuBisCO, and several others (Table 1). SF mitigates against the detrimental effect of PQ and enables plants to photosynthesize even when threated with PQ. None of these genes were repressed in SF-primed plants subsequently treated with PQ. On the contrary, a number of these genes had even higher levels of expression in the SF-primed plants, suggesting that SF actually induces their expression in both unstressed and PQ-stressed plants (Table 1). Upregulation in this manner may enhance photosynthetic efficiency in the SF-primed plants and also protect against stress, consistent with the healthy phenotype observed for all SF-treated groups. 

Gene *AT2G23670*, which encodes thylakoid protein YCF37, was upregulated in SF-primed plants compared to both PQ-stressed and untreated control plants. YCF37 is likely involved in photosystem 1 (PSI) assembly and oligomerization; its gene is induced by high-light stress and the protein has a likely role in repairing PSI during light stress [74]. A disrupted photosynthetic machinery and impaired photosynthesis result in the generation of various ROS, including superoxide radicals (O_2_^•–^) and singlet oxygen (^1^O_2_). Studies have also shown that to prevent high light-induced ^1^O_2_ accumulation and oxidative damage, it is essential to minimize the steady state concentration of chlorophyll biosynthetic intermediates in day light plants [75]. Here, we observed an increased level of *PROTOCHLOROPHYLLIDE OXIDOREDUCTASE C* (*PORC*) gene expression in SF-primed plants. The protein PORC is involved in chlorophyll biosynthesis. Under high-light conditions, transgenic *Arabidopsis* plants overexpressing PORC showed reduced accumulation of the chlorophyll biosynthesis intermediate protochlorophyllide (Pchlide), resulting in minimal ^1^O_2_ generation and protection from oxidative damage [75]. Increased expression of *PORC* in SF-primed plants may lead to a decrease in Pchlide, potentially protecting the plants from oxidative damage. Overall, transcriptomic analysis indicated that SF-treated plants likely maintain their photosynthetic machinery after PQ treatment and minimize ROS generation.

##### Carbohydrate Metabolism

Several genes related to carbohydrate metabolism were upregulated in the SF-primed plants (Table 1). *GALACTINOL SYNTHASE* (*GOLS*) encodes a key enzyme in the synthesis of raffinose family oligosaccharides (RFOs). Raffinose protect plants against PQ-induced oxidative stress [76]. Hence, upregulation of this pathway in SF-primed plants likely contributes to the enhanced tolerance to PQ observed in this study. 

Two other genes, *GLYCOSYL HYDROLASE 9C2*, also known as *ENDO-1,4-BETA-GLUCANASE 6*, and *TRICHOME BIREFRINGENCE-LIKE 27* (*TBL27*), were upregulated in SF-primed plants and may have functions in cellulose synthesis, cell wall deposition and O-acetylation of cell wall polysaccharides [77,78]. Glycosyl hydrolases are involved in cellulose biosynthesis, cell wall deposition and integration of polysaccharides [77]. O-acetylation of xyloglucans is possibly accomplished by the enzyme encoded by *TBL27* (also known as *ALTERED XYLOGLUCAN 4*, *AXY4*) [79]. Acetylation of cell wall polysaccharides is essential for maintaining the structural integrity of the leaf; it is also important for exerting a global impact on plant stress responses [80].

##### Growth and Hormone Signaling

Several growth-related genes are repressed by PQ, including genes encoding expansins (*EXPA8*, *EXPA11,* and *EXPB1*), auxin-responsive genes such as *IAA6*, *PIN7,* and *SAUR*-like genes. In contrast, these genes are not repressed in SF-PQ treated plants. On the contrary, many growth- and auxin-related genes are induced by SF in both PQ-stressed and unstressed SF-primed plants (Table 1). Gibberellin-regulated *GASA6* is also repressed by PQ but induced in SF-primed plants (stressed or unstressed), while *GIBBERELLIN 2-OXIDASE 6* (that inactivates GA) is induced only in PQ-treated unprimed plants. Taken together, these data show that while PQ represses growth, this effect of PQ is completely eliminated by SF. Furthermore, there is evidence that SF promotes growth (induction of growth-related genes as well as auxin and GA pathways), providing a molecular explanation of its action as a biostimulant/plant strengthener.

##### Autophagy

Oxidative stress can activate autophagy, an energy-dependent cellular process of recycling cellular components [81]. The WRKY33 transcription factor plays a role in regulating autophagy by interacting with autophagy-related (ATG) proteins [82,83]. Expression of *WRKY33* is highly induced by PQ while its transcript levels are even lower in SF-treated plants than controls (Table 1). Overexpression of *ATG8* (different genes including *ATG8E*) in *Arabidopsis* leads to an increase in the number of autophagosomes concomitant with an activated autophagy [84]. Similarly, experimental evidence was obtained to indicate that elevated expression of *ATG8H* increases autophagy during abiotic stress conditions [85]. Expression of both, *ATG8E* and *ATG8H* is repressed by SF (Table 1). Collectively, our observations suggest activation of autophagy by PQ and repression of autophagy by SF.

##### Lipid Metabolism

Genes related to lipid degradation including *PHOSPHOLIPASE A 2A* (*PLA2A*) and *MYZUS PERSICAE-INDUCED LIPASE1* (*MPL1*) [86] are strongly upregulated by PQ treatment, but not in PQ-treated plants previously primed with SF (Table 1). On the other hand, one gene encoding an oleosin-B3 like protein is downregulated by PQ treatment but upregulated in SF-primed plants. Oleosins are relatively small hydrophobic proteins (with a molecular weight of 15–26 kDa) associated with oil bodies, i.e., cellular organelles for the storage of triacylglycerols in plants. A knockout mutant of a gene coding oleosin-B3-like protein is hypersensitive to salt stress [87]. Lipid reconfigurations occur during oxidative stress balancing energy metabolism and mitigating the oxidative damage [23,88].

### 2.3. Metabolome Reconfiguration Induced by SF for Oxidative Stress Protection

**Figure 6:**
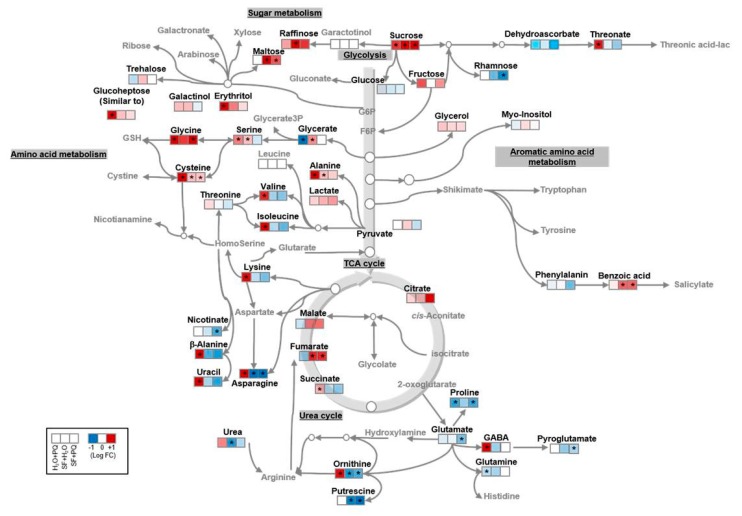
Primary metabolism pathways affected by oxidative stress in SF-primed and -unprimed plants. Changes in primary metabolite abundances in *Arabidopsis* leaves upon foliar spray with PQ and/or SF measured by GC-MS. Blue and red depict decreases and increases, respectively (log_2_ fold change), compared to the control (unprimed and unstressed, i.e., H_2_O + H_2_O). The data are averages of seven biological replicates. Samples: PQ-stressed (H_2_O+PQ), SF-primed, but unstressed (SF + H_2_O), and SF-primed and stressed (SF + PQ). Statistically significant differences compared to the control are highlighted by asterisks (at the significance level of 0.05, FDR corrected). Full data are given in Supplementary Materials Data S5.

To gain further insights into the effect of SF on plants, we determined the metabolic profiles under various conditions using GC-MS. We observed substantial differences in primary metabolites of oxidatively stressed plants when compared with untreated plants (Figure 5). Interestingly, application of SF prior to the PQ treatment almost completely abolished the metabolite changes observed in plants treated with only PQ (Figure 5). A detailed analysis of the metabolic profiles revealed an increase of threonate levels in PQ-treated plants compared to the untreated controls (H_2_O + PQ versus H_2_O + H_2_O), while threonate decreased in the other conditions (SF + PQ versus SF + H_2_O; Figure 5 and Figure 6). Threonate results from the degradation of ascorbate, with the ascorbate–glutathione cycle (Foyer–Halliwell–Asada pathway) representing an important system for ROS scavenging (Foyer and Noctor, 2011 [89]). The increase of threonate may suggest an impairment of the ascorbate–glutathione cycle for ascorbate regeneration in PQ-treated plants (Figure 5).

Interestingly, we observed an accumulation of various free amino acids in PQ-stressed plants, including branched chain amino acids (BCAAs), such as valine and isoleucine, as well as other amino acids such as alanine, glycine, cysteine, lysine, and asparagine. The amino acids most likely accumulate in the stressed plants as a result of protein degradation caused by PQ-induced disruption of regular metabolism rather than de novo amino acid biosynthesis, a conclusion supported by the fact that PQ causes cell death (Figure 1). Of note, an accumulation of amino acids during abiotic stresses has been observed in many different studies [21,90,91,92], and it has been suggested that metabolites accumulating under stresses might be used as building blocks for new cellular components to support recovery of growth after stress adaptation [21]. Moreover, one of the glutamate-derived compounds linked to oxidative stress responses is γ-aminobutyric acid (GABA) which is involved in pH regulation, stress responses, signaling, and other processes in plants [21,91,93]. Here, we observed that GABA over accumulates in PQ-stressed plants (Figure 5 and Figure 6). 

Metabolic and mechanical disruptions during stress cause cytosolic acidification and induce an acidic pH-dependent activation of GABA synthesis [93]. A stress-specific pattern of accumulation is consistent with a physiological role of GABA in stress mitigation [21,91,93,94,95]. Interestingly, some of the metabolic effects observed in PQ-stressed plants are not only characteristic of oxidative stress, but also occur in senescing plant tissues [96]. An important result obtained here is that the metabolic changes induced by PQ are almost completely abolished when plants are pre-treated with SF and then subjected to PQ treatment. Several TCA cycle intermediates, in particular, fumarate and, to some extent, citrate and malate, increased in SF-primed plants, effects which might support cellular respiration and photosynthesis [97,98]. It has previously been suggested that an accumulation of citrate under oxidative stress conditions may control the production of ROS and, therefore, increase the plant’s tolerance to stress [99,100]. Moreover, the levels of free amino acids were reduced following SF application which may indicate their utilization during the process of inducing stress tolerance and potentially growth (Figure 6). In contrast, raffinose accumulated after SF treatment (Figure 5 and Figure 6) which might increase oxidative stress tolerance as suggested previously [76]. The soluble sugar maltose accumulated in SF-primed and primed/stressed plants (Figure 5 and Figure 6). Maltose at physiologically relevant concentrations may protect proteins, membranes, and the photosynthetic electron transport chain from stress-induced damage [101].

### 2.4. Lipidome Level Changes Induced by SF for Oxidative Stress Protection

**Figure 7:**
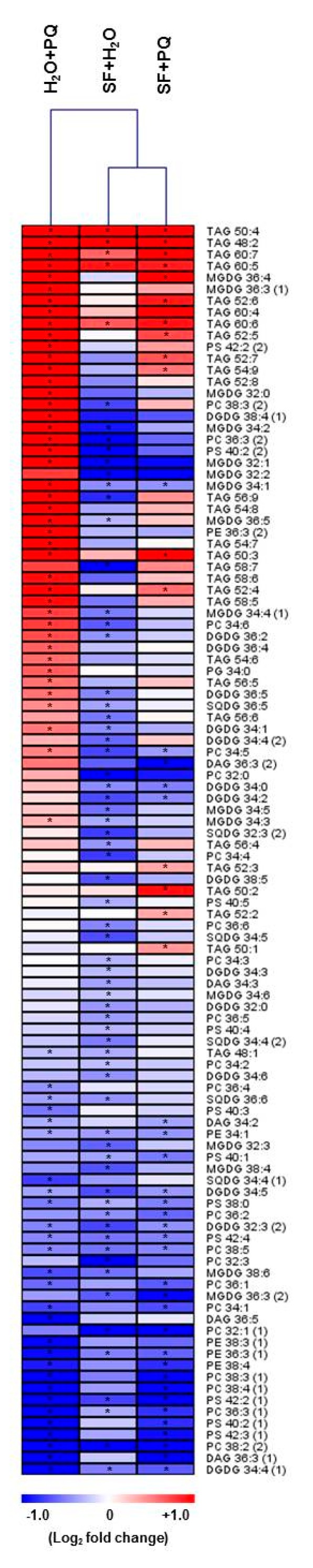
Lipid changes induced by oxidative stress. Changes in the abundances of lipids in *Arabidopsis* leaves after foliar spray with PQ and/or SF. Blue and red depict decreases and increases, respectively, compared to the control (unprimed and unstressed, i.e., H_2_O + H_2_O). Data are given in log_2_ fold change; only lipids significantly different from control samples are shown (highlighted by an asterisk). The data are averages of seven biological replicates. Samples: PQ-stressed (H_2_O + PQ), SF-primed but unstressed (SF + H_2_O), and SF-primed and stressed (SF + PQ). Statistically significant differences to control are highlighted by asterisks (at the significance level of 0.05, FDR corrected). Full data are given in Supplementary Materials Data S6. DAG: diacylglycerol; TAG: triacylglycerol; MGDG: monogalactosyldiacylglycerol; DGDG: digalactosyldiacylglycerol; PC: phosphatidylcholine; PE: phosphatidylethanolamine; PG: phosphatidylglycerol; PI: phosphoinositol; PS: phosphoserine; and SQDG: sulfoquinovosyldiacylglycerol.

Lipids are the main components of biological membranes and they represent important carbon and energy storage compounds in plants [102,103]. Lipids may sense extracellular signals and trigger lipid-mediated signaling and have also been shown to accumulate during several stresses, e.g., when photosynthesis is inhibited [102,103]. Here, we observed major changes in lipid profiles of plants exposed to oxidative stress (Figure 7). In total, 155 annotated lipids from ten neutral and polar lipid classes were identified. Of those, 108 lipids were significantly changed in at least one of the tested conditions in comparison with the untreated control condition (Figure 7). In our analysis, we detected different classes of lipids including neutral lipids of the diacylglyceride (DAG) and triacylglyceride (TAG) classes, as well as polar lipids including digalactosyldiacylglycerol (DGDG), monogalactosyldiacylglycerol (MGDG), phosphatidylcholine (PC), phosphatidylethanolamine (PE), phosphatidylglycerol (PG), and sulfoquinovosyldiacylglycerol (SQDG). It has been shown that under adverse conditions, neutral lipids play an important role in intracellular homeostasis and energy balance [104].

A more detailed analysis of the 108 significantly changed lipids revealed that most of the neutral TAG lipids and polar chloroplast lipids, such as, for example, galactolipids (i.e., MGDG and DGDG) which constitute the bulk of the chloroplast membrane, increased in stressed plants, likely resulting from cell death and chloroplast degradation, while they were primarily decreased in SF-treated plants (Figure 7).

Notably, the TAG class of neutral lipids (representing the main source of energy) showed divergent patterns in different conditions: the levels of some lipids (e.g., TAG 60:5 and TAG 60:6), increased while levels of others (e.g., TAG 54:0 and TAG 54:2) decreased in PQ-stressed plants. A contrasting pattern was observed in SF-primed plants. A large proportion of the observed alterations in lipid levels in stressed plants was reversed in stressed plants pre-treated with SF, which demonstrates the protective role of SF against oxidative stress. Most of the neutral lipids, namely, TAGs and DAGs, decreased when stressed plants were compared with stressed plants pre-treated with SF (Figure 7). This observation is in line with previous reports which showed that plants under adverse stress condition, such as, for example, extended darkness, usually accumulate those lipid classes [88], an effect mitigated by application of SF, as shown here.

### 2.5. Assessment of the Relationship between Molecular and Phenotypic Changes Induced by SF

**Figure 8:**
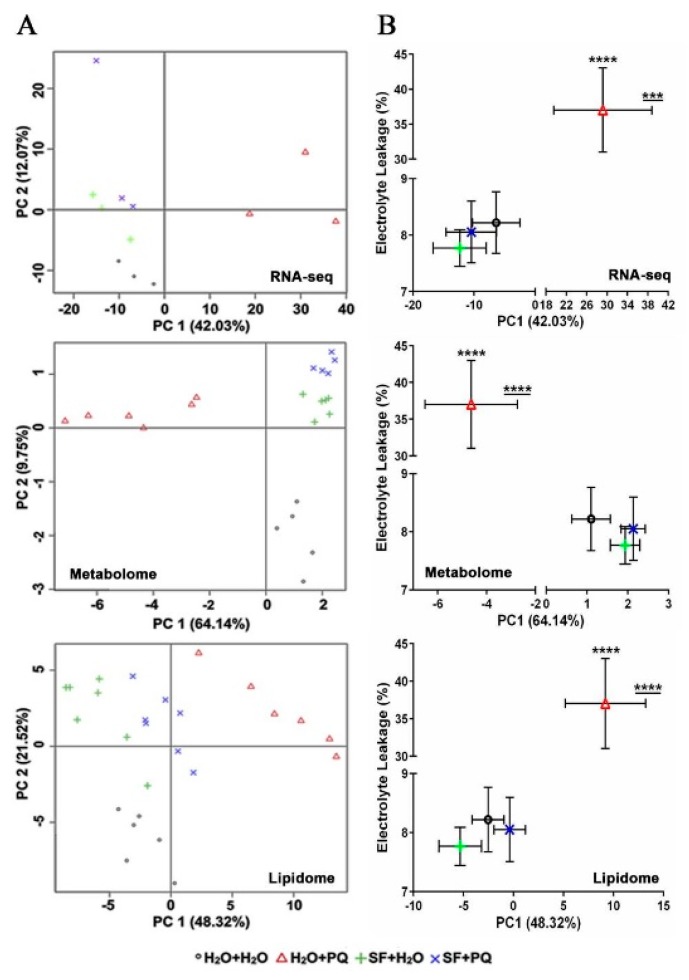
Principle component analysis (PCA) of omics data. (**A**) PCA of RNA-seq, metabolome, and lipidome data. Plotted are three biological replicates per condition for RNA-seq, five to six biological replicates per condition for metabolome, and seven biological replicates per condition for lipidome. Samples: unprimed and unstressed (H_2_O + H_2_O), PQ-stressed (H_2_O + PQ), SF-primed but unstressed (SF + H_2_O), and SF-primed and stressed (SF + PQ). (**B**) Scatter plot of PC1 values versus mean electrolyte leakage (%). Mean PC1 values were compared among groups using one-way ANOVA followed by Tukey’s multiple comparisons test to correct for multiple testing. For both, PC1 and electrolyte leakage, a significant difference was observed between the H_2_O + PQ group (oxidatively stressed) and each of the three remaining groups. For PC1, the adjusted *p*-values for all three pairwise comparisons were *p* ≤0.0004 (***), <0.0001 (****), and <0.0001 (****) for transcriptome, metabolome, and lipidome, respectively (underlined in the graph; see Figure S2 for comparison of PC1 values by one-way ANOVA).). Horizontal error bars denote the standard deviation (SD) of PC1 values. Vertical error bars indicate the SD of electrolyte leakage. Full data are given in Supplementary Materials Data S1 and S7.

Principal component (PC) analysis (Figure 8A) demonstrates that a high level of variation in the transcriptome (42.03%), metabolome (64.14%), and lipidome (48.32%) among treatment conditions can be explained by the first component (PC1). In all cases, three of the treatment conditions (i.e., unprimed and unstressed (H_2_O + H_2_O), SF-primed and unstressed (SF + H_2_O), SF-primed and stressed (SF + PQ)) cluster closely together along the first component (PC1). In contrast, the unprimed but PQ-stressed group (H_2_O + PQ) was found to deviate significantly from the other treatment groups in all the omics approaches used (Figure 8A). This result indicates a higher similarity of H_2_O- and SF-treated plants at the molecular level and suggests that SF treatment induces stress adaptations by inhibiting damaging stress responses such as those induced by PQ. Furthermore, the electrolyte leakage data show a similar trend to the mean PC1 values, supporting the earlier observation (Figure 8B). This was not observed for PC2 (Figure S2 and Figure S3). Finally, the results of the PCA were strongly correlated to the visible plant phenotypes obtained (Figure 1): the two SF-treated groups are phenotypically similar to the negative control with which it clusters along PC1, whilst the oxidatively damaged plants are distant from that cluster. Thus, many of the molecular changes induced by SF and PQ manifest phenotypically.

## 3. Materials and Methods 

### 3.1. Plant Material, Stress Treatments, and RNA Extraction

*Arabidopsis thaliana* (Columbia-0) seeds were sown in soil and the pots were kept for four days for vernalization (4 °C in darkness) for uniform seed germination. Thereafter, the pots were kept in a growth chamber in a 12 h light (150 μE m^−2^ s^−1^, 20 °C, 60% relative humidity)/12 h dark (16 °C, 70% relative humidity) cycle. At least 30 uniformly developed 23 day-old plants were used for the experiments. The *Ascophyllum nodosum* seaweed extract, SuperFifty (SF), was produced by and obtained from BioAtlantis Ltd. (Tralee, County Kerry, Ireland). For physiological and molecular analyses, multiple applications of 0.1% SF were performed, three times a day, starting three hours after day light by two-hour intervals, on two consecutive days. This was followed by 15 µM PQ foliar treatment, 24 h after the last SF application. Uniform spraying was achieved by spraying from a 10 cm distance and one spray/plant—the approximate volume of each spray was around 750 µL. Rosette leaves were harvested 24 h after the PQ treatment from three biological replicates for phenotypic and transcriptome analyses and from seven biological replicates for primary metabolite and lipidome analyses. Total RNA was extracted from rosette leaves of the treated plants and controls by RNeasy Plant Mini Kit (Qiagen, Hilden, Germany). Each biological replicate was a pool of at least two rosette leaves. 

### 3.2. Electrolyte Leakage Measurements and Trypan Blue Staining 

Electrolyte leakage measurements were performed at the rosette stage by checking conductivity with a HI 8733 conductivity meter (Hanna Instruments, Woonsocket, RI, USA) as described in Reference [23]. Visualization of dead cells was done using detached rosette leaves employing a slightly modified version of trypan blue staining protocol explained in Reference [105]. In short, entire detached leaves were incubated for 30 min in hot trypan blue staining solution (80–90 °C). Thereafter, leaves were de-stained by rinsing them once in water, followed by incubation in 15 M chloral hydrate for two hours. The de-staining solution was renewed when needed (two–three times) before taking photos. 

### 3.3. Transcriptome Sequencing and Data Analysis 

Transcriptome sequencing was performed by LGC Genomics (Berlin, Germany) using the Illumina HiSeq 4000 platform to obtain 75 bp-long single-end reads. The quality of raw reads was evaluated with FastQC (http://www.bioinformatics.babraham.ac.uk/projects/fastqc). Sequencing adaptors were trimmed using Cutadapt [106], and reads below 20 bp length were discarded. Ribosomal RNA contamination was assessed using SortMeRNA (v2.1) [107], and reads aligning to rRNA were filtered out. Processed reads were quantified using kallisto (v0.43.0; bootstraps: 100) [108] against *Arabidopsis* cDNA sequences (Araport11) [109] to obtain gene expression levels. Differential expression analysis was carried out using EdgeR package in R/Bioconductor [110]. The FDR cutoff < 0.001 and absolute log_2_ fold change ≥ 1 were used to identify significantly differentially expressed genes. Generation of heatmaps and clustering of genes were performed with the pheatmap R-package [111], using k-means clustering. GO enrichment of Biological Process terms and KEGG pathway enrichment analyses were carried out using GOSeq [112] and clusterProfiler [113], respectively, with FDR cutoff < 0.05. The enriched GO terms and KEGG pathways were plotted using ggplot2 R-package [114].

Sequencing data are available from the NCBI Bioproject database (www.ncbi.nlm.nih.gov/bioproject) under ID PRJNA526343.

### 3.4. Gas Chromatography-Mass Spectrometry (GC-MS) Analysis of Primary Metabolites 

GC-MS was employed for the analysis of primary metabolites. Primary metabolites from 50 (±5) mg ground freeze-dried leaf material were extracted following extraction and derivatization procedure as described [115]. Peaks were annotated manually, and ion intensity was determined by the aid of TagFinder software [116], using a reference library from the Golm Metabolome Database [117] and following the recommended reporting format [118]. The relative abundances were normalized to fresh weight of relevant samples and were plotted as heatmap by Multiple Experiment Viewer (MeV; http://mev.tm4.org).

### 3.5. Lipid Profiling 

Lipids were extracted from 50 (±5) mg ground freeze-dried leaf material using the methyl tert-butyl ether (MTBE) method [119]; data were analyzed as reported [23]. Raw lipid intensities were log_2_ transformed to bring them closer to normal distribution and to exclude the dominant effect of extreme small/large values [120]. The heatmap of fold changes was drawn using MeV.

### 3.6. Analysis of Differential Behavior of Metabolites and Lipids

Analysis of differential behavior was conducted on the data after the preprocessing, described above. Differential behavior between pairwise conditions (i.e., SF + PQ versus H_2_O + H_2_O, SF + H_2_O versus H_2_O + H_2_O, and H_2_O + PQ versus H_2_O + H_2_O) was inspected using a linear model. We applied the R-package limma [121]. Metabolites and lipids were considered as differentially behaving under a certain condition (SF + PQ, SF + H_2_O, or H_2_O + PQ) if their levels were significantly different from the control condition (H_2_O + H_2_O) at the significance level of 0.05 (FDR corrected). 

### 3.7. Principal Component Analysis

Principal component analysis (PCA) from the R package pcaMethods [122] was applied to the log_2_ transformed metabolite/lipid data; outlier samples were removed. For transcriptome data, the log_2_ transformed expression levels were used for PCA analysis using the prcomp function in the R package stats.

### 3.8. Statistical Analysis

Statistical analysis for electrolyte leakage and PCA analysis was performed by GraphPad Prism 6.0 (GraphPad Software, San Diego, CA, USA) using one-way ANOVA followed by Tukey’s multiple comparisons test to adjust the *p*-values.

## 4. Conclusions

Our transcriptome, metabolome, and lipidome analysis provided a system-wide level understanding of the protective role induced by the *Ascophyllum nodosum* biostimulant SF against oxidative stress in *Arabidopsis*. Comparative transcriptomics identified oxidative stress mechanisms affected by SF priming. Transcription factors and genes linked to stress adaptation mechanisms, such as autophagy and reactive oxygen detoxification, were identified. Moreover, upregulation of photosynthesis, hormone signaling, and growth-related genes provided evidence for stimulation of growth induced by SF. Increased levels of several primary metabolites in SF-primed plants, including maltose and raffinose, suggest a contribution to stress protection. Lipid profiling revealed that alterations in lipids were associated with reduction of cell death and chloroplast degradation and enhancement of intracellular energy balance in SF-primed plants during oxidative stress. Collectively, our data reveal that treatment of plants with SF prior to a stress may be employed as a climate-smart strategy to alleviate oxidative stress-induced damages in crops.

## Figures and Tables

**Table 1:** SuperFifty and paraquat alter the expression of genes representing fundamental biochemical pathways and physiological processes. Values are TMM (trimmed mean of M-values) values averaged across three biological replicates.

	Average TMM Values				Annotation	
Gene ID	H_2_O + H_2_O	H_2_O + PQ	SF + H_2_O	SF + PQ	Gene Name	Description/Function
**ROS Inducible/Marker genes**						
AT2G21640	19.63	97.21	16.99	20.09		Marker for oxidative stress response protein
AT2G43510	64.24	214.79	40.04	47.11	*TI1*	Trypsin inhibitor, ROS marker
AT1G57630	4.07	76.13	4.36	4.51		Toll-Interleukin-Resistance (TIR) domain family protein
AT1G19020	36.57	247.44	26.24	33.40		CDP-diacylglycerol-glycerol-3-phosphate 3-phosphatidyltransferase, ROS marker
AT1G05340	8.55	83.32	6.72	5.41		Cysteine-rich stress protein, ROS marker
**ROS-Dependent PCD and Senescence**						
AT4G25110	1.75	6.47	2.07	2.99	*MC2*	Metacaspase 2
AT1G16420	0.15	5.83	0.37	0.47	*MC8*	Metacaspase 8
AT5G02190	2.78	2.35	7.33	8.40	*PSC1*	Promotion of cell survival 1
AT5G02760	11.95	3.34	24.69	16.34	*SSPP*	Senescence-suppressed protein phosphatase
AT4G27410	73.77	100.20	27.15	22.12	*RD26*	NAC (No Apical Meristem) domain transcriptional regulator superfamily protein
AT2G29350	31.09	395.89	19.27	16.05	*SAG13*	Senescence-associated gene 13
**ROS Detoxification**						
AT4G25100	459.32	277.99	664.81	715.19	*FSD1*	Fe superoxide dismutase 1
AT4G09010	378.15	197.56	405.53	403.35	*APX4*	Ascorbate peroxidase 4
AT5G57345	207.99	168.40	323.34	337.82	*OXR*	Transmembrane OXR protein
AT4G39800	90.77	59.99	143.09	125.49	*MIPS1*	Myo-inositol-1-phosphate synthase 1
AT5G21100	23.74	13.69	30.65	24.71	*AO*	Plant L-ascorbate oxidase
AT5G51720	90.38	74.87	122.85	149.99	*NEET*	2 iron, 2 sulfur cluster binding protein
AT2G45560	43.67	26.91	67.23	67.88	*CYP76C1*	Cytochrome P450 family 76C, polypeptide 1
AT2G46660	1.07	1.12	2.12	2.39	*CYP78A6*	Cytochrome P450, family 78A, polypeptide 6
AT3G26310	7.03	4.62	11.01	9.27	*CYP71B35*	Cytochrome P450, family 71B, polypeptide35
AT4G12320	32.62	28.68	66.06	74.88	*CYP706A6*	Cytochrome P450, family 706A, polypeptide6
**Photosynthesis**						
AT5G38420	1223.01	567.26	1376.75	1518.00	*RBCS2B*	RuBisCO small chain
AT3G27690	799.90	303.31	1142.16	1032.88	*LHCB2.3*	Photosystem II LHC protein 2.3
AT1G51400	1851.61	886.74	2086.64	2069.37		Photosystem II 5 kD protein
AT3G08940	3027.19	1449.15	3533.29	3026.97	*LHCB4.2*	Light harvesting complex photosystem II
AT3G63160	2922.20	1632.49	3304.18	3817.24	*OEP6*	Outer envelope membrane protein
AT3G27690	799.9	303.31	1142.16	1032.88	*LHCB2.4*	Chlorophyll a-b binding protein 2.4
AT2G34430	3293.32	1182.9	3677.36	2958.81	*LHB1B1*	Light harvesting chlorophyll protein complex II subunit B1
AT3G08940	3027.19	1449.15	3533.29	3026.97	*LHCB4.2*	Chlorophyll a-b binding protein CP29.2
AT1G51400	1851.61	886.74	2086.64	2069.37		Photosystem II 5kD protein
AT2G34430	187.98	155.10	290.55	340.92	*LHB1B1*	LHC II subunit B1
AT2G23670	187.98	155.10	290.55	340.92		YCF37
AT1G03630	183.08	99.92	212.62	230.60	*PORC*	Protochlorophyllide oxidoreductase C
**Carbohydrate Metabolism and Cellulose Synthesis**						
AT1G64390	59.50	35.02	96.35	99.01		Glycosyl hydrolase 9C2
AT2G01290	13.39	18.80	28.15	33.23		Ribose-5-phosphate isomerase 2
AT1G70230	8.11	9.73	16.53	15.36		Trichome Birefringence-Like 27
AT1G09350	24.01	8.36	45.35	36.17		Galactinol synthase 3
**Growth and Hormone Signaling**						
AT1G21310	32.88	197.79	33.87	31.90	*EXT3*	Extensin 3
AT1G76930	73.22	149.21	53.68	60.34	*EXT4*	Extensin 4
AT1G20190	59.51	26.15	93.85	83.53	*EXPA11*	Expansin 11
AT2G20750	7.22	3.03	14.97	15.91	*EXPB1*	Expansin B1
AT2G40610	50.54	18.78	99.07	79.61	EXPA8	Expansin A8
AT5G57560	77.60	111.40	28.68	21.14	*TCH4*	Xyloglucan endotransglucosylase/hydrolase
AT2G14620	1.10	9.39	0.72	0.99	*XTH10*	Xyloglucan endotransglucosylase/hydrolase 10
AT3G44990	29.15	26.58	73.92	99.89	*XTH31*	Xyloglucan endo-transglycosylase-related 8
AT2G21210	25.55	15.99	53.74	65.71	*SAUR6*	SAUR-like auxin-responsive protein family
AT4G38860	27.84	11.74	56.72	52.80	*SAUR16*	SAUR-like auxin-responsive protein family
AT1G75580	4.29	4.93	8.13	10.95	*SAUR51*	SAUR-like auxin-responsive protein family
AT4G38840	97.43	43.53	134.62	150.76	*SAUR14*	SAUR-like auxin-responsive protein family
AT4G38850	9.36	3.92	15.98	15.53	*SAUR15*	SAUR-like auxin-responsive protein family
AT3G53250	1.95	0.68	4.90	3.47	*SAUR57*	SAUR-like auxin-responsive protein family
AT1G23080	65.86	32.20	94.83	82.05	*PIN7*	Auxin efflux carrier family protein
AT2G46870	4.70	3.63	9.38	8.47		AP2/B3-like TF, auxin response.
AT5G13320	3.71	16.08	2.97	3.60	*PBS3*	Auxin-responsive GH3 family protein
AT4G12550	4.01	1.69	5.66	6.45	*AIR1*	Auxin-Induced in Root cultures 1
AT1G52830	4.59	1.53	6.79	4.16	*IAA6*	Indole-3-acetic acid 6
AT1G74670	150.45	33.60	188.25	141.53	*GASA6*	Gibberellin-regulated family protein
AT1G02400	7.18	18.48	5.67	5.18	*GA2OX6*	Gibberellin 2-oxidase 6 (inactivates gibberellin)
AT1G15550	8.09	2.49	5.96	6.32	*GA3OX1*	Gibberellin 3-oxidase 1
**Autophagy**						
AT2G38470	48.75	142.40	26.84	36.51	*WRKY33*	WRKY DNA-binding protein 33
AT2G45170	82.62	98.94	38.58	32.66	*ATG8E*	AUTOPHAGY 8E
AT3G06420	18.68	35.54	14.28	14.12	*ATG8H*	AUTOPHAGY 8H
**Lipid Metabolism**						
AT1G19020	36.57	247.44	26.24	33.40		CDP-diacylglycerol-glycerol-3-phosphate 3-phosphatidyltransferase
AT4G34200	75.62	216.87	76.14	74.59	*EDA9*	D-3-phosphoglycerate dehydrogenase
AT4G39670	3.43	50.87	2.08	2.82		Glycolipid transfer protein (GLTP) family protein
AT1G67800	10.05	23.91	10.77	12.32		Copine (Calcium-dependent phospholipid-binding protein) family
AT3G55470	18.33	57.91	20.04	21.00		Ca-dependent lipid-binding (CaLB domain)
AT5G14180	1.77	5.23	0.84	0.62	*MPL1*	*Myzus persicae*-induced lipase 1
AT2G26560	45.59	364.62	38.48	42.54	*PLA2A*	Phospholipase A 2A
AT1G13930	2740.50	1898.62	4318.3	3952.83		Oleosin-B3 like protein
AT1G51080	18.67	11.26	25.10	27.68		Golgin family A proteins
AT1G25054	0.61	0.57	6.08	3.07	*LPXC3*, *LPXC4*	UDP-3-*O*-acyl N-acetylglycosamine deacetylase
**Transcription Factors and Stress-Related Genes**						
AT1G59930	4.7	2.6	8.94	10.58		MADS-box transcription factor
AT1G70890	136.76	85.38	171.01	170.27	*MLP43*	MLP-like protein 43
AT1G75690	184.17	98.65	215.40	223.10		DnaJ/Hsp40
AT1G78070	43.04	33.13	77.16	73.50		Transducin/WD40
AT2G28720	95.98	74.93	131.89	151.73	*HTB3*	Histone super family protein
AT2G41090	300.65	461.43	464.31	652.82	*CML10*	Calcium-binding EF-hand family protein
AT2G44940	7.88	6.32	20.17	28.83		Integrase-type DNA-binding

## Supplementary Materials

Supplementary materials can be found at https://www.mdpi.com/1422-0067/21/2/474/s1. Figure S1: Expression profiles of all 20 clusters obtained from k-means clustering using heatmap, Figure S2: Comparison of mean PC1 and PC2 values among treatment groups, Figure S3: Scatter plot of PC2 values versus mean electrolyte leakage (%), Table S1: Number of differentially expressed genes for each pairwise comparison, Table S2: List of 35 DEGs significantly upregulated upon SF-priming versus untreated control, Data S1: Data of Figure 1C and Figures S1 and S3, Data S2: Data of Figure 4 and Figure S1, Data S3: Data of Figure 2 and Figure 3, Data S4: Expression of known ROS marker genes, Data S5: Data of Figure 5 and Figure 6, Data S6: Data of Figure 7, Data S7: Data of Figure 8, Figures S2 and S3.
