# Supplementary figures and images for: A Biostimulant Obtained from the Seaweed Ascophyllum nodosum Protects Arabidopsis thaliana from Severe Oxidative Stress
Source: Int J Mol Sci. 2020 Jan 11;21(2):474. doi: 10.3390/ijms21020474 (PMC7013732; doi:10.3390/ijms21020474)

**Figure S1**

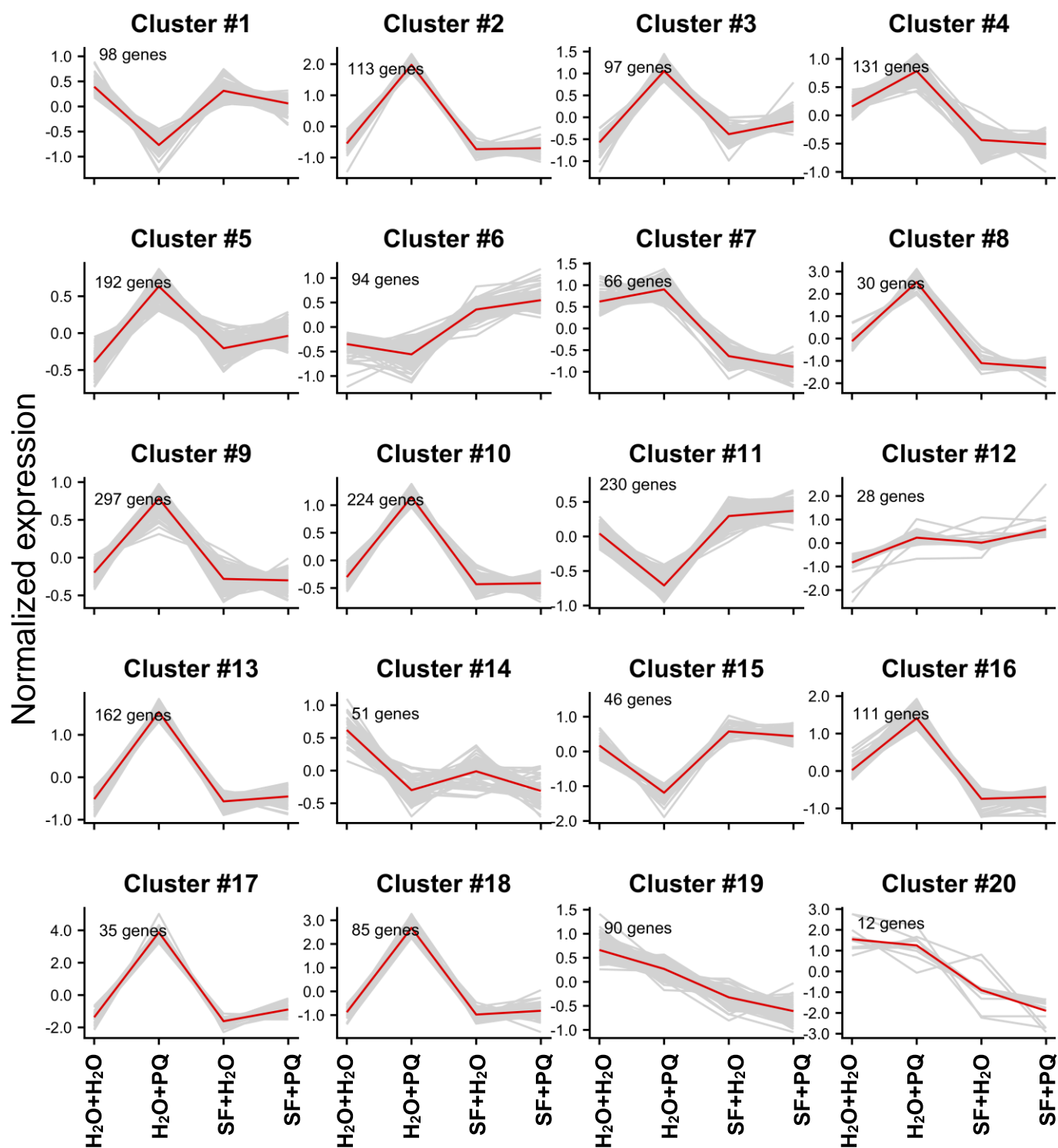

**Figure S2**

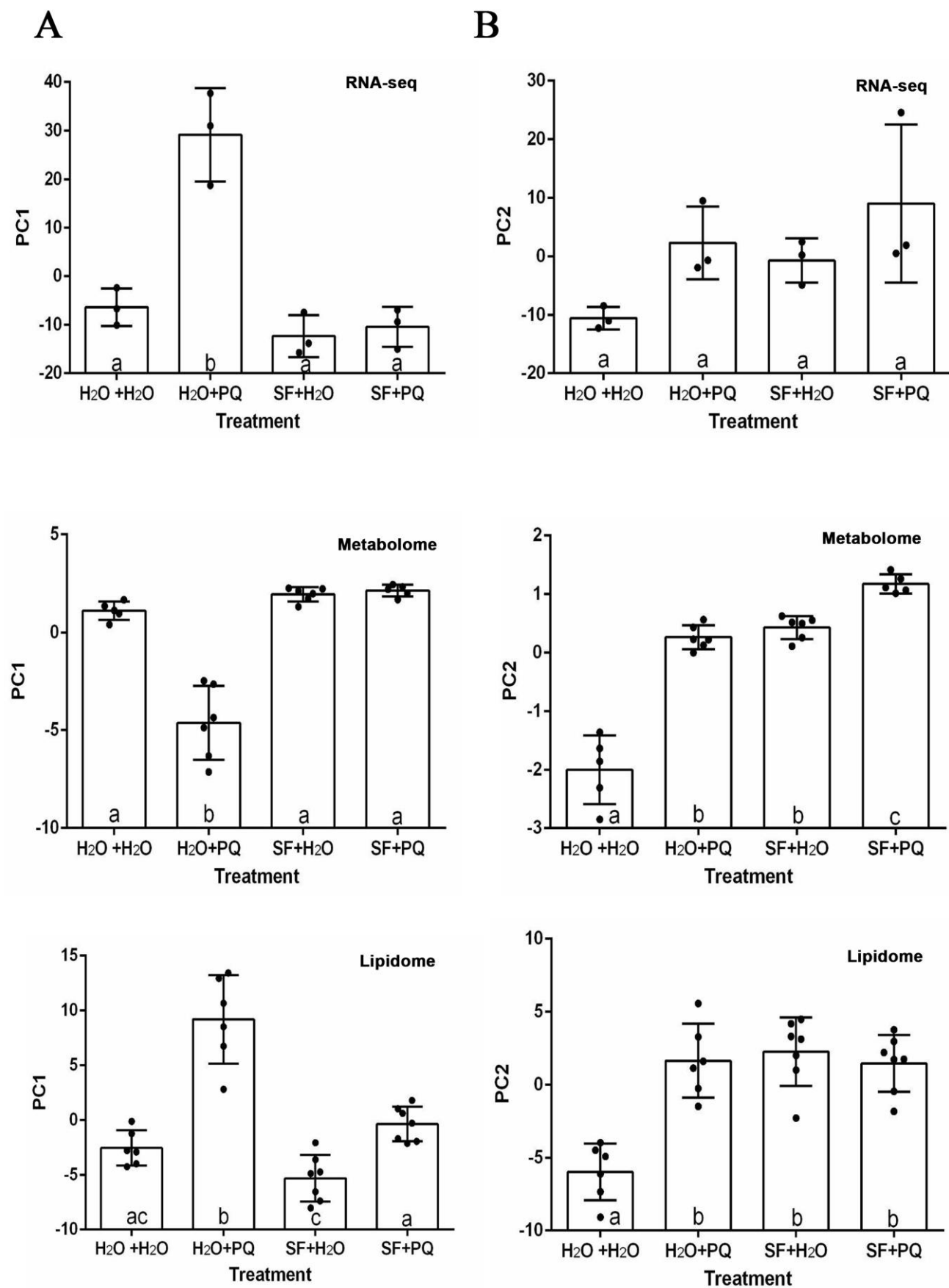

**Figure S3**

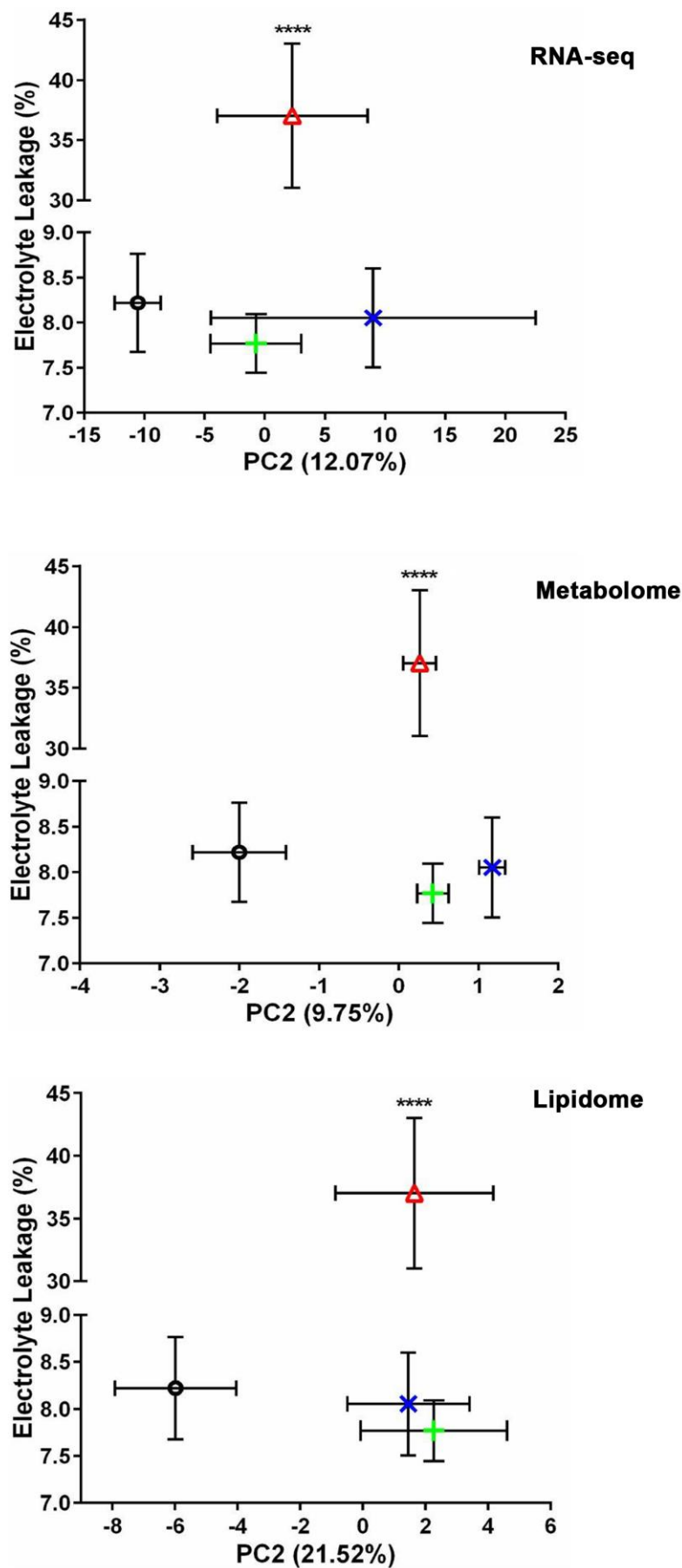

Supplement: Supplementary file 1 [file ijms-21-00474-s001.zip › Supplementary Figures.pdf]

## Slide 1
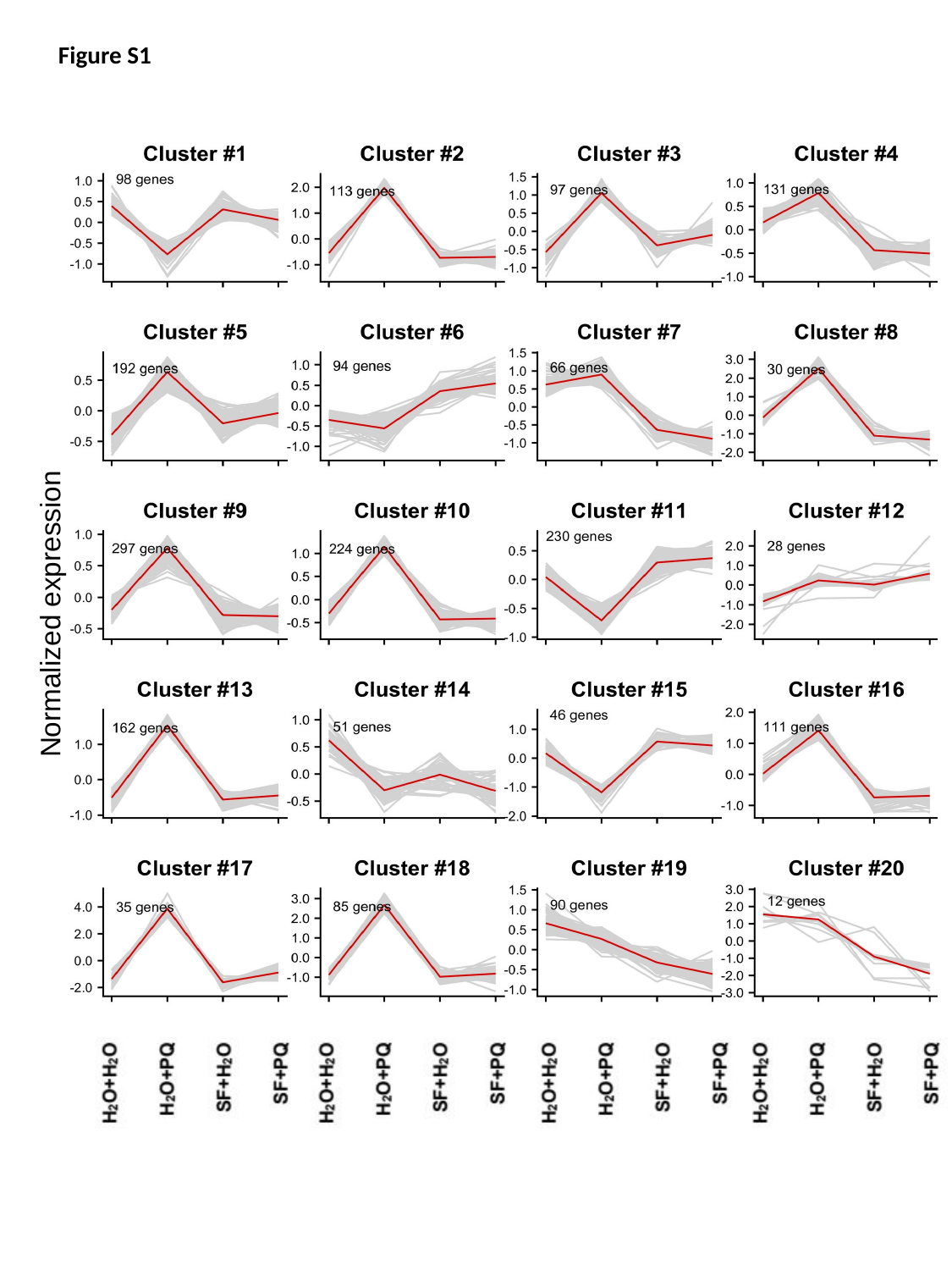

Figure S1
Normalized expression

## Slide 2
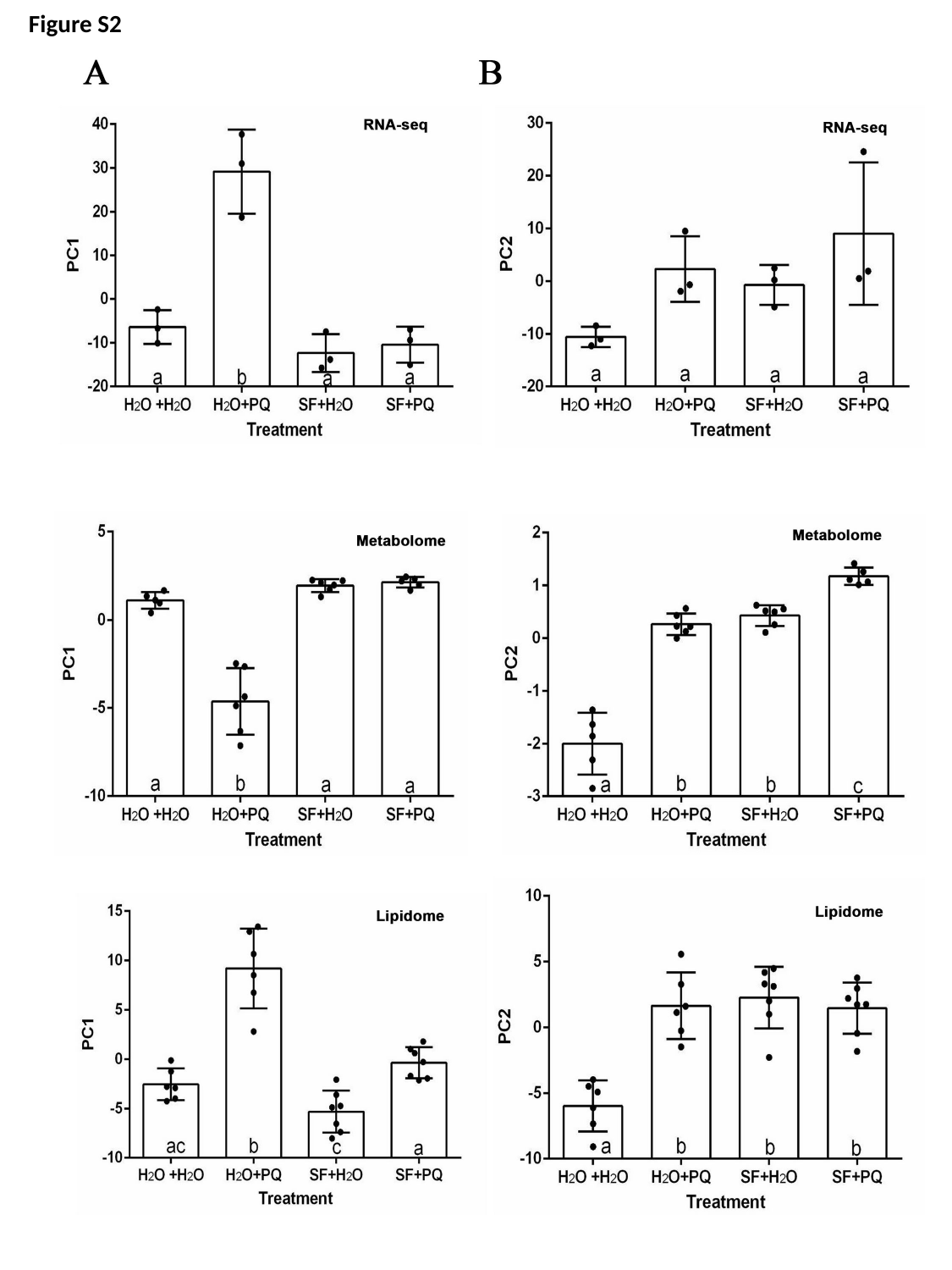

Figure S2

## Slide 3
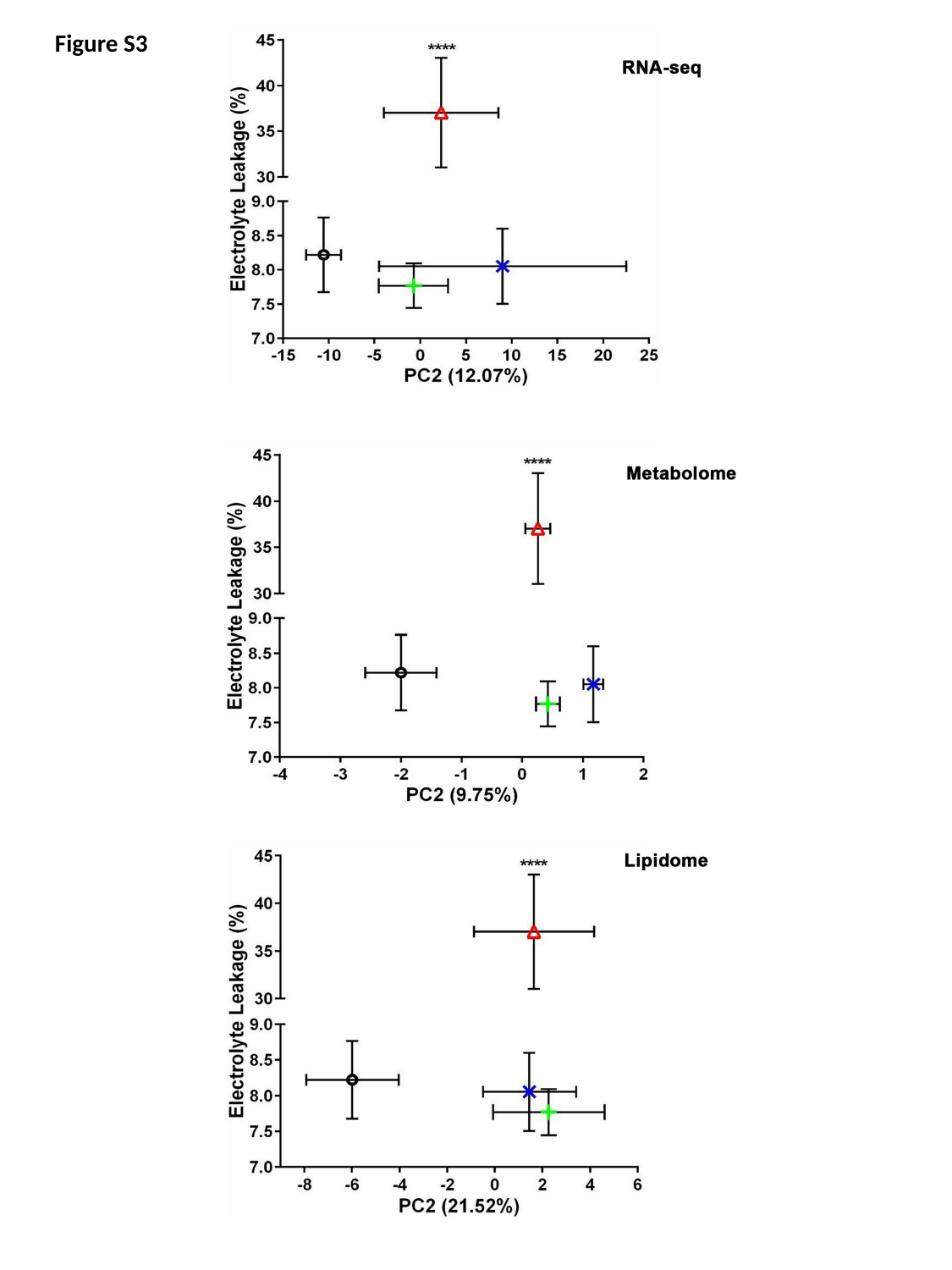

Figure S3

Supplement: Supplementary file 1 [file ijms-21-00474-s001.zip › Supplementary Figures.pptx]
